# Teaching treatment planning for protons with educational open‐source software: experience with FoCa and matRad

**DOI:** 10.1002/acm2.12326

**Published:** 2018-05-12

**Authors:** Daniel Sanchez‐Parcerisa, Jose Udías

**Affiliations:** ^1^ Grupo de Física Nuclear Departamento de Estructura de la Materia Física Térmica y Electrónica & UPARCOS Universidad Complutense de Madrid & CEI Moncloa Madrid Spain; ^2^ Instituto de Investigación Sanitaria del Hospital Clínico San Carlos (IdISSC) Madrid Spain

**Keywords:** education, medical physics, proton therapy, teaching, treatment planning

## Abstract

Open‐source, MATLAB‐based treatment planning systems FoCa and matRAD were used in a pilot project for training prospective medical physicists and postgraduate physics students in treatment planning and beam modeling techniques for proton therapy. In the four exercises designed, students learnt how proton pencil beams are modeled and how dose is calculated in three‐dimensional voxelized geometries, how pencil beam scanning plans (PBS) are constructed, the rationale behind the choice of spot spacing in patient plans, and the dosimetric differences between photon IMRT and proton PBS plans. Sixty students of two courses participated in the pilot project, with over 90% of satisfactory rating from student surveys. The pilot experience will certainly be continued.

## 1 INTRODUCTION

A Treatment Planning System, or TPS, is a special type of software specifically designed for creating, evaluating, administering, and archiving radiotherapy treatments. The key features of any TPS are the ability to calculate three‐dimensional dose distributions on a CT of the patient and the capacity to optimize the plan to match the prescription of the oncologist. TPS must undergo a thorough calibration and commissioning process before they become suitable for clinical use. For the sake of robustness, and to protect the code from potentially unsafe alterations, commercial TPS tend to have a closed architecture. Furthermore, due to intellectual property protection the source code of those systems is rarely disclosed. Because of this, commercial TPS are neither widely available nor easily affordable outside clinical environments, which limits the possibilities of offering education on treatment planning to university hospitals (able to spare a share of their clinical TPS licenses for educational purposes, often reserved to their own residents), professional schools organized by national or international societies such as ESTRO or AAPM (targeting mostly current residents or practicing medical physicists), or software‐specific seminars organized by the vendors themselves (inevitably biased toward a certain solution).

This problem has already been discussed in the context of conventional radiotherapy,^1^, ^2^ but it becomes much more relevant for proton and carbon ion therapy, simply because of the smaller number of centers treating patients. Hospitals offering residency programs, even those considering the installation of a proton therapy solution, have a hard time training their own staff in the specifics of planning for proton and carbon ion therapy due to the lack of available planning software.

To overcome this difficulty and facilitate research and teaching activities, two MATLAB‐based treatment planning systems, FoCa 3 and matRad^4^ were developed, respectively, at the University of Pennsylvania and the German Center for Cancer Research (DKFZ). They come to join other in‐house created TPS that achieved clinical maturity, such as Plan‐UNC^5^ (not available for hadron therapy), ASTROID,^6^ or TriP98,^7^ which were developed to fill gaps where no commercial system was available, but they have become invaluable educational tools at the institutions where they were created.

After being used successfully in several research projects,^8^, ^9^, ^10^, ^11^, ^12^, ^13^ FoCa and matRad were utilized for teaching purposes in two courses during academic year 2016/2017 at the our University: Nuclear Physics Applied to Medicine (from the MSc in Nuclear Physics) and Hadron therapy (from the Summer School on Advanced Topics in Medical Physics), with a total of over 60 students. The objective of the learning experiences was the familiarization of the students with treatment planning techniques, pencil beam modeling, and radiobiology applied to treatment planning. FoCa, with more complex beam models and analytical LET calculation, was the code of choice for studying the physical properties of the beam, while matRad was used to illustrate radiobiological concepts, such as relative biological effectiveness (RBE), and to illustrate the difference in dose distributions produced by clinical proton and photon beams.

## 2 MATERIALS AND METHODS

The students, divided into pairs, carried out several small projects including the determination of the optimal set of parameters for a clinical proton beam and the planning of a clinical case. The sessions were held at the main informatics classroom of the Faculty of Physics, where students remotely connected via secure shell to the high‐capacity cluster of the Faculty, with 64 GB of memory in the main node and a total of 8 nodes and 64 cores. MATLAB R2013b, compatible with both FoCa and matRad, was installed in the cluster using the floating license scheme of the university. Both codes were downloaded and installed from their respective online repositories, http://nuclear.fis.ucm.es/foca and https://github.com/e0404/matRad.

For the FoCa exercises, students were given commented MATLAB script files (.m) with detailed information on how to fill in certain gaps to complete the exercise. For the matRad experiences, students were asked to load one of the phantoms freely distributed with the code and perform certain operations on it as directed by their instructor.

## 3 EXERCISES AND DIDACTIC VALUE

### 3.1 Experience with FoCa

**Figure 1:**
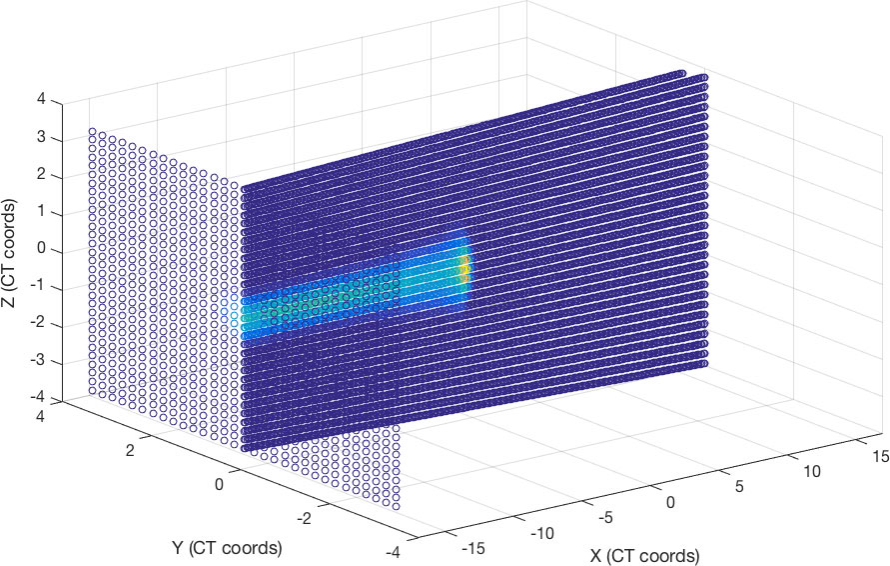
Scheme of the dose calculation grid in FoCa with an example proton plan with a single monoenergetic pencil beam.

**Figure 2:**
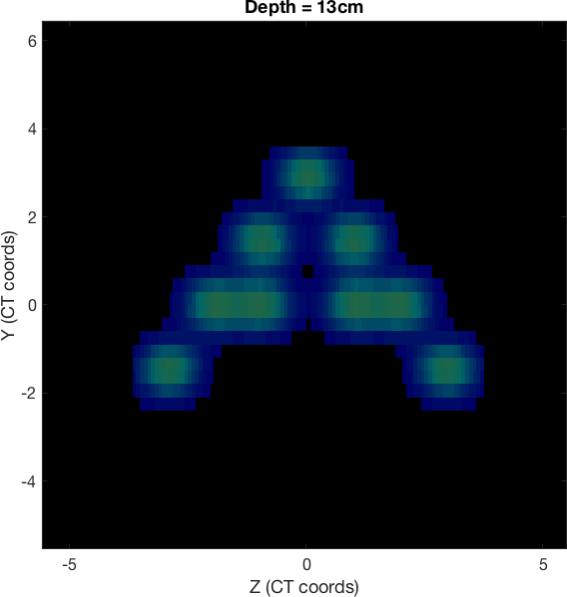
Transversal cut of a dose cube irradiated by a proton therapy plan with nine spots forming the shape of letter A.

The experience was divided into three exercises. In the first one, students were asked to load a predefined example plan (with a single, monoenergetic proton pencil beam) and draw the beam position and a set of transversal and longitudinal dose distributions (Figure 1). This helped students familiarize with the coordinate system and characteristics of the pencil beam. Then, the class was asked to complete the plan with more spots, forming a certain letter of the alphabet in the transversal dose distribution (Figure 2). This practice was aimed at showing how different beam spots are placed together to conform a single proton field.

**Figure 3:**
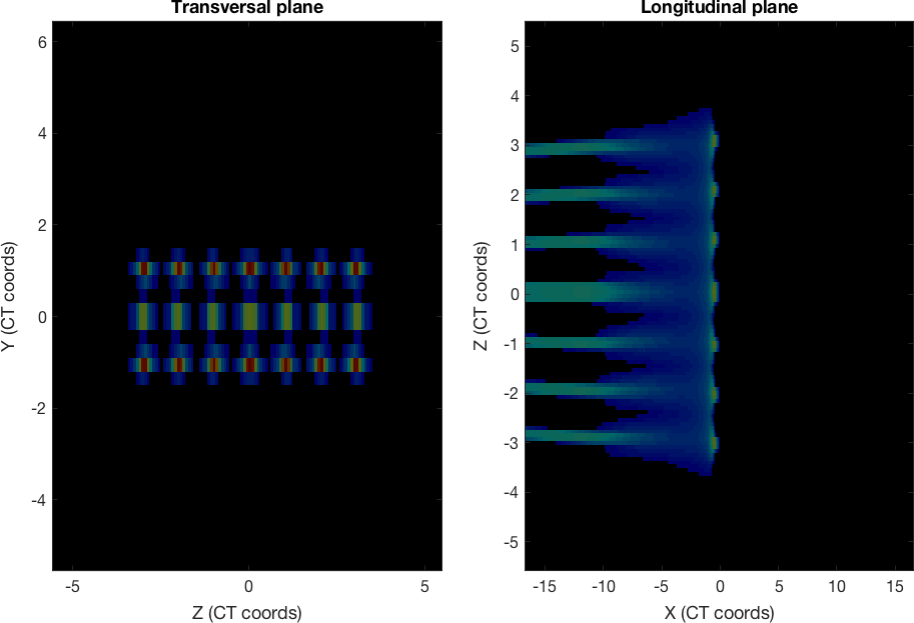
Transversal and longitudinal cuts for a set of proton beams in a water cube.

**Figure 4:**
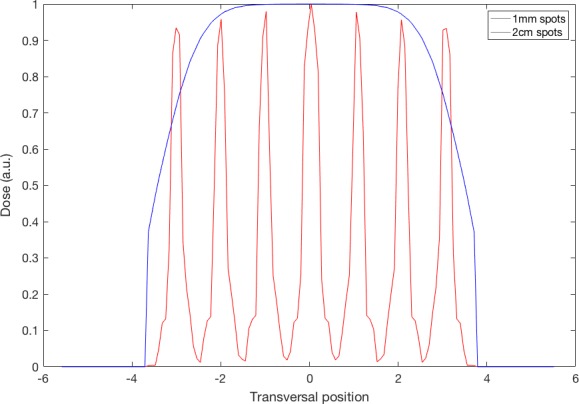
Transversal beam profiles for sharp (1‐mm sigma) and broad (2‐cm sigma) proton beams with a separation of 1 cm, measured at the peak position, placed at the isocenter.

In the second exercise (see Figure 3), the class studied how the spot size and beam spacing influence the shape of the field in a pencil beam scanning proton plan. If the spots are small or are too spread apart, the field will not be homogeneous; if the spots are too large, the lateral penumbra will increase beyond clinically acceptable limits. Starting from a single‐layer plan with 24 spots (distributed in three rows of eight spots) and a given spot size at the isocenter, students were asked to find the optimal spacing between spots to minimize the lateral penumbra while keeping the field homogeneity (in the isocenter plane) within certain limits (Figure 4). The didactic value of this experience lies on the identification of the key physical pencil beam parameters responsible for the characteristics of the proton fields.

**Figure 5:**
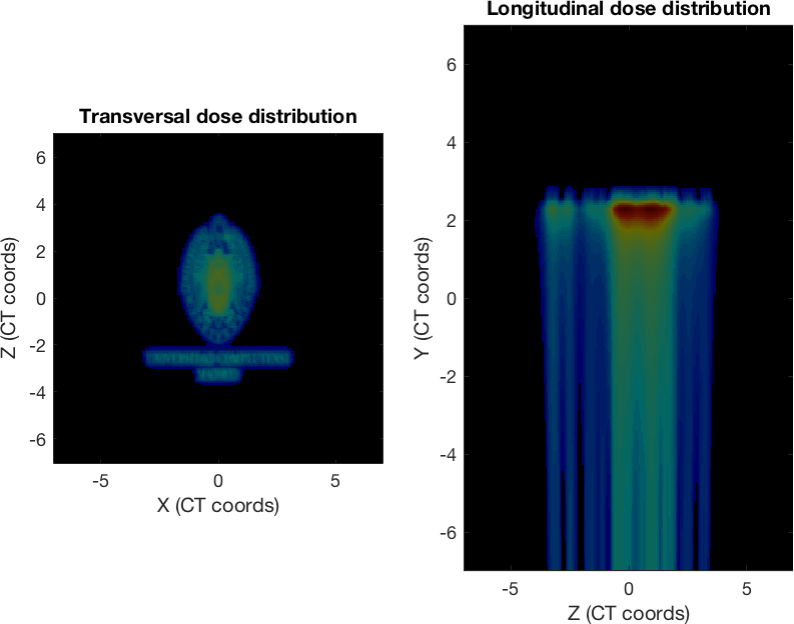
Dose calculation of a monoenergetic proton plan forming the logo of our University using 1‐mm sigma spots.

The last FoCa exercise was designed to showcase the possibilities of proton radiation. Starting from a homogeneous spot distribution on a single‐layer plan, the task was to load an image and write a simple script to modify the beam weights of the plan to dose‐paint the selected image. Figure 5 shows an example plan, created from the logo of the University.

### 3.2 Experience with matRad

**Figure 6:**
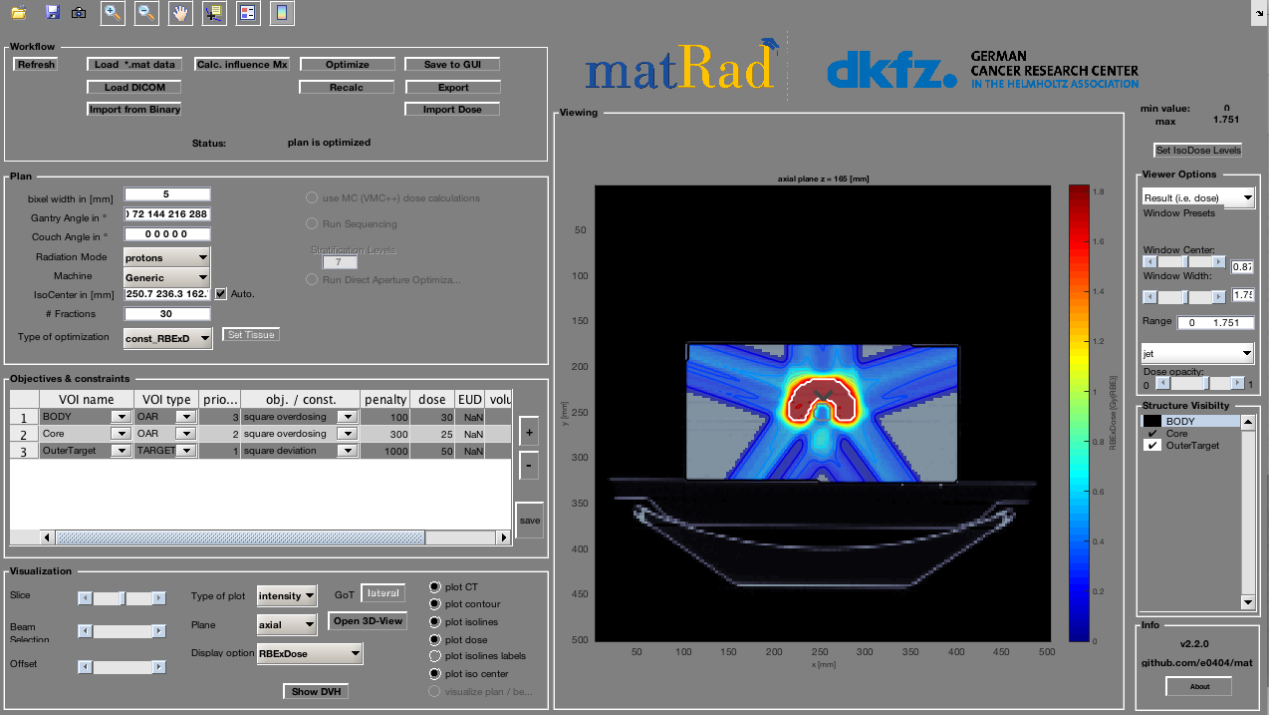
Snapshot of matRad graphical user interface performing a radiobiological optimization for a five‐field proton plan on the TG119 Phantom.

While FoCa is more of a research code than a full TPS, matRad does have a complete graphical user interface resembling a commercial system, as well as optimization capabilities (see Figure 6). Additionally, it includes not only proton beams but also carbon ion therapy and IMRT with photon beams. It is therefore an ideal candidate for showcasing the capabilities of hadron therapy in terms of better dose conformality.

In the proposed exercise, students loaded an anonymous patient CT, corresponding to a prostate tumor case, and performed two different plans on it with the same constraints: a five‐field IMRT plan (beam orientations with gantry angles 0°, 72°, 144°, 216°, and 288° in IEC coordinates), and a two‐field parallel‐opposed proton plan (PBS), with gantry angles at 90° and 270°. After optimization, they were asked to compare the resulting dose–volume histograms (DVHs) and to answer questions relative to homogeneity of the dose distributions and irradiation of surrounding organs, namely femoral heads, rectum, and bladder.

## 4 CONCLUSION AND DISCUSSION

This pilot project has demonstrated how, using freely available open‐source projects, it is possible to give an initial training to prospective medical physicists (not yet registered on a residency program) on the specificities of proton treatment planning without investing in commercial TPS programs. The experience allowed the instructors to demonstrate concepts explained during theoretical lectures in medical physics and to put in contact out students with novel techniques in research and clinical radiation therapy. More than 90% of the students that took part in the pilot project rated it “satisfactory” or above in end‐of‐term surveys, with no remarkable differences in the ratings given to FoCa and matRad exercises. The students improved their understanding of the material and their ability to actively assimilate the course content and therefore, the experiences will certainly be continued in subsequent courses.

Some concerns were raised about the fact that, being FoCa and matRad open‐source codes, both rely on a commercial platform such as MATLAB. While these concerns are valid, the use of MATLAB is widespread in research and university environments and its availability is by no means comparable to that of commercial radiotherapy treatment planning systems.

Finally, some instructors and students reported having issues related to computation speed with both codes, with certain calculations and optimizations taking more than 15/20 min to complete. These issues were caused by concurrent sharing of resources (memory and computational power) between students. Since treatment planning is an inherently computationally demanding problem, with computing requisites (particularly, in terms of memory) slightly above the capacity of an average general‐usage computer, the planning of the exercises must account for the available resources and limit, where necessary, the number of students per session, to prevent such computational bottlenecks to have a negative impact in the learning experience.

## ACKNOWLEDGMENTS

The authors acknowledge support from the Spanish Government (FPA2013‐41267, FPA2015‐65035‐P, RTC‐2015‐3772‐1, XIORT IPT‐2012‐0401‐300000), Comunidad de Madrid (S2013/MIT‐3024 TOPUS‐CM), European Regional Funds, and the Moncloa Campus of International Excellence (“Grupo de Física Nuclear‐UCM”). Ref. 910059. Part of the calculations of this work were performed in the “Clúster de Cálculo para Técnicas Físicas” funded in part by UCM and in part by UE Regional Funds. This work acknowledges partial support by EU's H2020 under MediNet as a networking activity of ENSAR2 (Grant Agreement 654002). The authors also thank the students who participated in the pilot projects, for their valuable feedback.

## CONFLICT OF INTEREST

The authors declare no conflicts of interest to report.
